# Supplementary material for: Epigenetic Age Acceleration in Frontotemporal Lobar Degeneration: A Comprehensive Analysis in the Blood and Brain
Source: Cells. 2023 Jul 24;12(14):1922. doi: 10.3390/cells12141922 (PMC10378390; doi:10.3390/cells12141922)
Supplement: Supplementary file 1 [file cells-12-01922-s001.zip › cells-2430573-supplementary.pdf]

Epigenetic Age Acceleration in Frontotemporal Lobar Degeneration: A Comprehensive Analysis in the Blood and Brain

Megha Murthy [1,2], Patrizia Rizzu [3], Peter Heutink [3,4], Jonathan Mill [5], Tammaryn Lashley [1,6] and Conceição Bettencourt [1,6,*]

[1] Queen Square Brain Bank for Neurological Disorders, UCL Queen Square Institute of Neurology,
London WC1N 1PJ, UK; t.lashley@ucl.ac.uk (T.L.)
[2] Department of Clinical and Movement Neurosciences, UCL Queen Square Institute of Neurology,
London WC1N 1PJ, UK
[3] German Center for Neurodegenerative Diseases (DZNE), 72076 Tübingen, Germany
[4] Alector, Inc., South San Francisco, CA 94080, USA
[5] Department of Clinical and Biomedical Sciences, Faculty of Health and Life Sciences, University of Exeter, Exeter EX4 5DW, UK
[6] Department of Neurodegenerative Disease, UCL Queen Square Institute of Neurology, London WC1N 1PJ, UK
* Correspondence: c.bettencourt@ucl.ac.uk

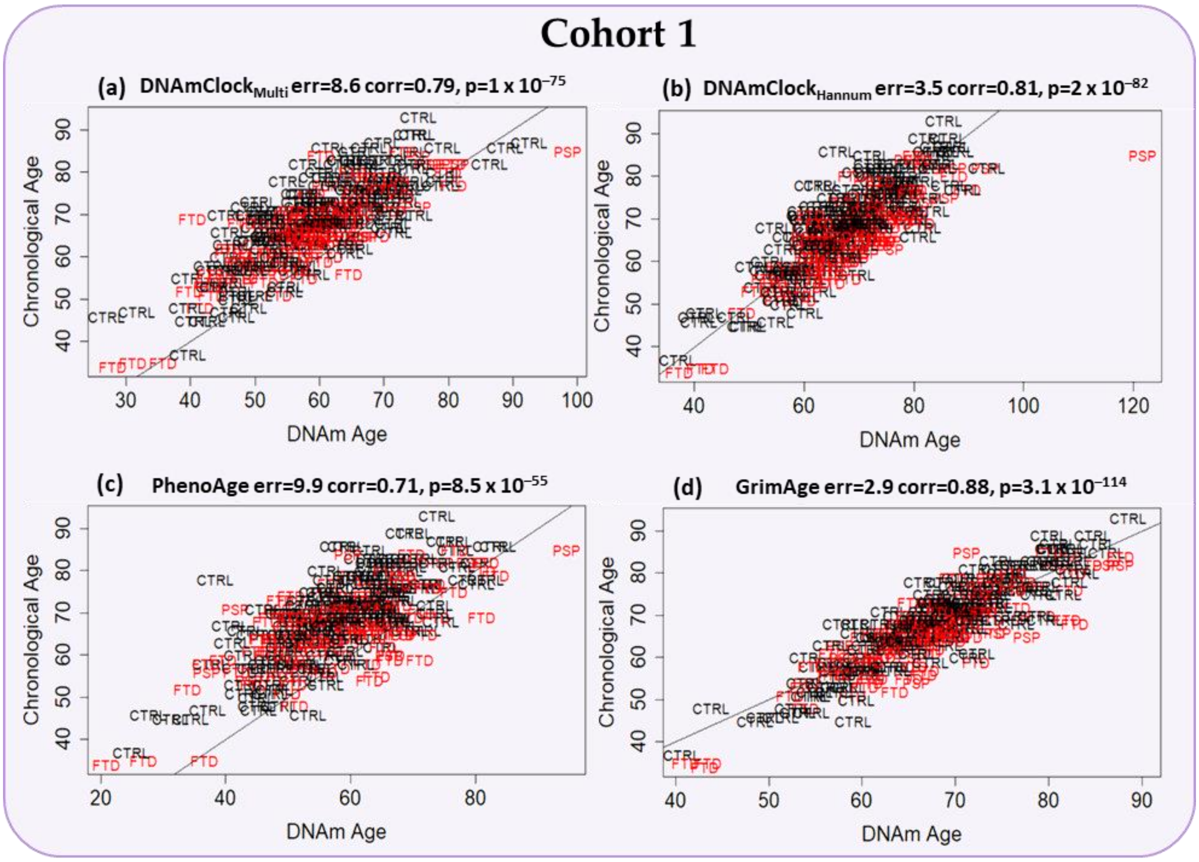

Figure S1. Epigenetic age analysis of the peripheral blood samples in Cohort 1 (purple) with the DNAmClock$_{Multi}$, DNAmClock$_{Hannum}$, PhenoAge, and GrimAge clocks: cohort 1 constituted FTD (n=117) and PSP cases (n=44) as well as controls (n=178). (**a–d**) Correlations between chronological age (y-axis) and DNAm age (x-axis) for all 4 clocks. CTRL – control, FTD – frontotemporal dementia, PSP – progressive supranuclear palsy.

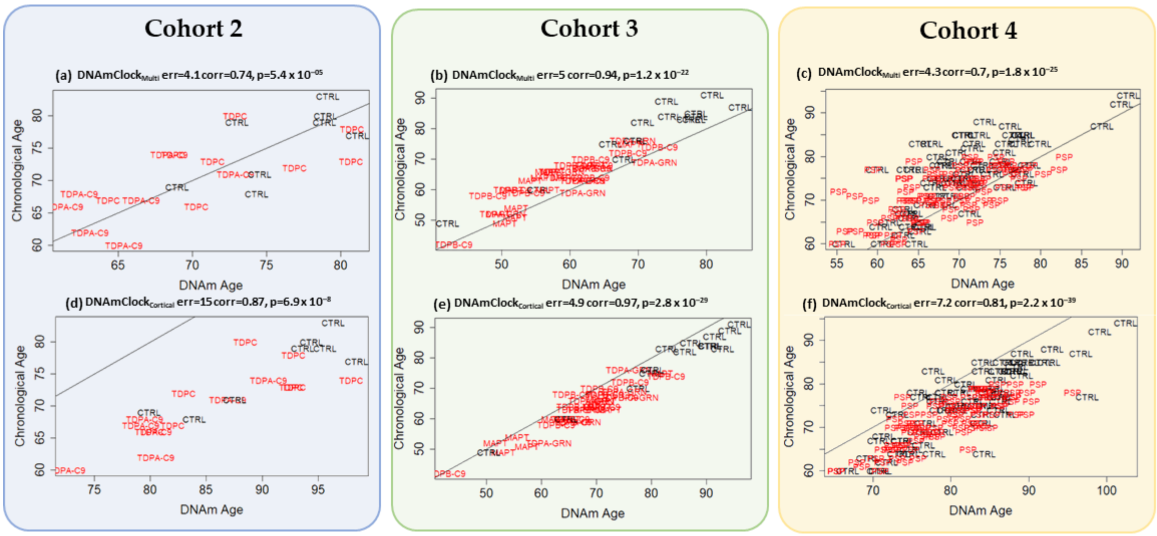

Figure S2. Epigenetic age analysis of post-mortem brain tissues using DNAmClock$_{Multi}$ and DNAmClock$_{Cortical}$: cohort 2 (blue) constituted FTLD-TDP types A (*C9orf72* mutation carriers, n=7), and C (sporadic cases, n=8), and controls (n=8), Cohort 3 (green) constituted FTLD-TDP types B (*C9orf72* mutation carriers n=13), and A (*GRN* mutation carriers, n=7), FTLD-Tau *MAPT* mutation carriers (n=13) and controls (n=14), and Cohort 4 (yellow) comprised PSP cases (n=93) and controls (n=71). (**a–f**) Correlations between chronological age (y-axis) and DNAm age (x-axis) for the cohorts with DNAmClock$_{Multi}$ and DNAmClock$_{Cortical}$. CTRL – control, TDPA-C9 – FTLD-TDPA (*C9orf72* mutation carriers), TDPC – FTLD-TDPC (sporadic); TDPA-GRN – FTLD-TDPA (*GRN* mutation carriers), TDPB-C9 – FTLD-TDPB (*C9orf72* mutation carriers), MAPT – FTLD-Tau *MAPT* mutation carriers, PSP – progressive supranuclear palsy.

Table S1. Average DNAm ages and average age acceleration for the different cohorts.

A) Peripheral Blood

**Sample Group**	**Average DNAm age, years (SD)**				**Average age acceleration (residuals)**						
**Cohort 1**	**DNAmClock$_{Multi}$**	**DNAmClock$_{Hannum}$**	**PhenoAge**	**GrimAge**	**DNAmClock$_{Multi}$**	**DNAmClock$_{Hannum}$**	**PhenoAge**	**GrimAge**	**IEAA$_{Multi}$**	**IEAA$_{Hannum}$**	**EEAA**
Controls	59.8 (10.5)	68.1 (10.1)	58.4 (10.6)	59.8 (10.5)	-0.5	-1.2	-0.9	-0.6	-0.5	-0.8	-1.6
FTD	57.1 (9.3)	66.7 (8.5)	57.4 (10.8)	57.1 (9.3)	0.3	**0.8***	1.1	0.6	0.4	**0.7***	**1.1***
PSP	63.9 (9.4)	73.9 (10.5)	59.6 (10.0)	63.9 (9.4)	1.6	**3.0***	-0.1	0.8	1.0	**1.7***	**3.7***
Total	59.4 (10.1)	68.3 (9.9)	58.2 (10.6)	59.4 (10.1)							

B) Post-mortem brain tissue

**Sample Group**	**Average DNAm age, years (SD)**		**Average age acceleration (residuals)**	
**Cohort 2**	**DNAmClock$_{Multi}$**	**DNAmClock$_{Cortical}$**	**DNAmClock$_{Multi}$**	**DNAmClock$_{Cortical}$**
Controls	76.1 (4.1)	91.2 (6.6)	1.3	0.0
FTLD-TDPA (*C9orf72*)	65.8 (4.0)	81.4 (5.8)	-2.2	-0.6
FTLD-TDPC (Sporadic)	73.2 (5.8)	88.7 (6.4)	0.6	0.5
Total	72.0 (6.3)	87.3 (7.3)		
**Cohort 3**				
Controls	71.3 (11.2)	83.2 (12.6)	-0.2	-0.2
FTLD-TDPA (*GRN)*	62.4 (7.2)	70.3 (5.9)	2.5	1.1
FTLD-TDPB (*C9orf72)*	59.4 (8.3)	67.8 (9.3)	0.1	-0.5
FTLD-Tau (*MAPT)*	56.0 (5.5)	65.5 (8.9)	-0.9	0.1
Total	62.4 (10.3)	72.1 (12.2)		
**Cohort 4**				
Controls	70.7 (6.6)	82.3 (735)	-0.5	-0.5
PSP	68.6 (6.3)	79.7 (6.1)	0.4	0.4
Total	69.5 (6.5)	80.8 (6.9)		

Cohort 1 – purple; cohort 2 – blue; cohort 3 – green; cohort 4 – yellow; FTD – frontotemporal dementia; PSP – progressive supranuclear palsy; FTLD – frontotemporal lobar degeneration; FTLD-TDPA/C – FTLD with 43 kDa transactive response DNA-binding protein (TDP-43) positive inclusions, types A and C; *C9orf72* – *C9orf72* mutation carriers; *GRN* – *GRN* mutation carriers, FTLD-Tau – FTLD with tau-positive inclusions; *MAPT* – *MAPT* mutation carriers, SD – standard deviation, IEAA – intrinsic epigenetic age acceleration; EEAA – extrinsic epigenetic age acceleration. *** Significant age acceleration upon pairwise comparisons with the corresponding controls.**

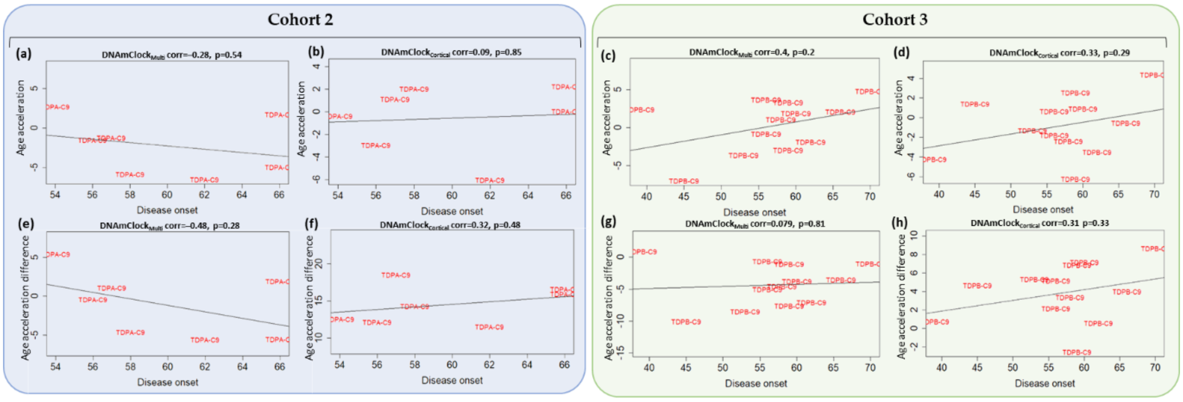

Figure S3. Association between age acceleration and disease onset with DNAmClock$_{Multi}$ and DNAmClock$_{Cortical}$ for the *C9orf72* mutation carriers in cohorts 2 (blue) and 3 (green): age acceleration residuals (y-axis) for DNAmClock$_{Multi}$ and DNAmClock$_{Cortical}$ versus disease onset (x-axis) for (**a**,**b**) Cohort 2 (FTLD-TDPA *C9orf72* mutation carriers), and (**c**,**d**) Cohort 3 (FTLD-TDPB *C9orf72* mutation carriers), age acceleration difference (y-axis) versus disease onset (x-axis) for (**e**,**f**) Cohort 2 and (**g**,**h**) Cohort 3. Age acceleration residuals were obtained by regressing DNA methylation age against chronological age and adjusting for confounding factors such as neuronal proportions obtained using a DNA methylation-based cell-type deconvolution algorithm. Age acceleration difference was the difference between DNA methylation age and chronological age. The correlation coefficient and p-values shown were calculated using Pearson correlation. TDPA-C9 - FTLD-TDPA (*C9orf72* mutation carriers), TDPB-C9 - FTLD-TDPB (*C9orf72* mutation carriers).

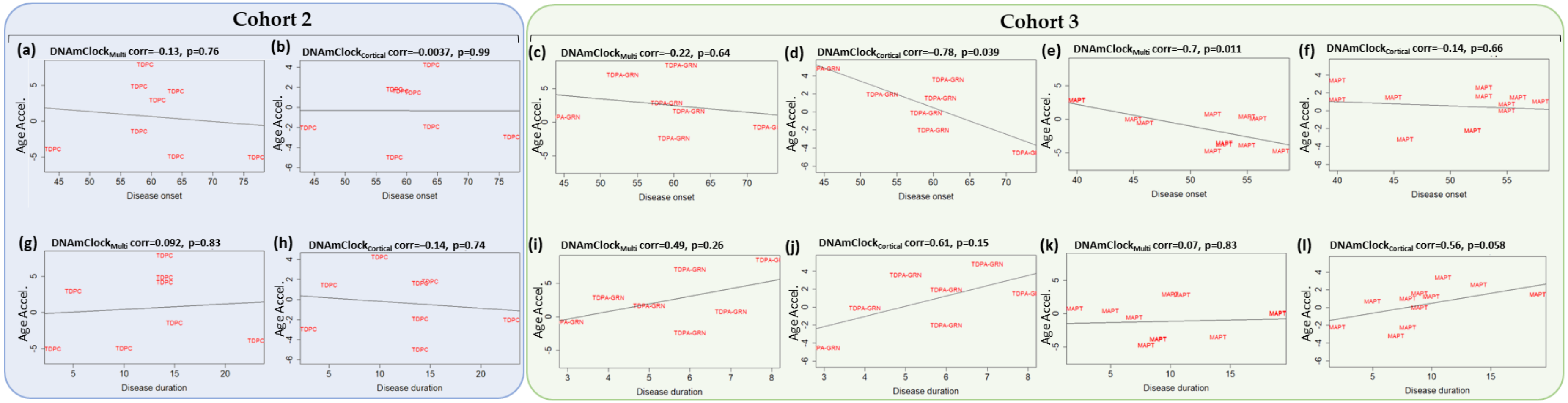

Figure S4. Association between age acceleration and disease onset and duration with DNAmClock$_{Multi}$ and DNAmClock$_{Cortical}$ for the FTLD-TDPC subtype in cohort 2 (blue) and FTLD-TDPA-*GRN* and FTLD-Tau-*MAPT* mutation carriers in cohort 3 (green): Age acceleration residuals (y-axis) for DNAmClock$_{Multi}$ and DNAmClock$_{Cortical}$ versus disease onset (x-axis) for (**a**,**b**) Cohort 2 (FTLD-TDPC), and (**c–f**) Cohort 3 (*GRN* and *MAPT* mutation carriers). Age acceleration residuals (y-axis) for DNAmClock$_{Multi}$ and DNAmClock$_{Cortical}$ versus disease duration (x-axis) for (**g**,**h**) Cohort 2 (FTLD-TDPC), and (**i–l**) Cohort 3 (*GRN* and *MAPT* mutation carriers). Age acceleration residuals were obtained by regressing DNA methylation age against chronological age and adjusting for confounding factors such as neuronal proportions obtained using a DNA methylation-based cell-type deconvolution algorithm. The correlation coefficient and p-values shown were calculated using Pearson correlation. TDPC – FTLD-TDP subtype C, TDPA-GRN – FLTD-TDPA *GRN* mutation carriers, MAPT – FTLD-Tau *MAPT* mutation carriers.
